# Correction: Direct measurement of the mechanical work during translocation by the ribosome

**DOI:** 10.7554/eLife.04563

**Published:** 2014-09-04

**Authors:** Tingting Liu, Ariel Kaplan, Lisa Alexander, Shannon Yan, Jin-Der Wen, Laura Lancaster, Charles E Wickersham, Kurt Fredrick, Harry Noller, Ignacio Tinoco, Carlos J Bustamante

Liu T, Kaplan A, Alexander L, Yan S, Wen JD, Lancaster L, Wickersham CE, Fredrick K, Noller H, Tinoco I Jr, Bustamante CJ. 2014. Direct measurement of the mechanical work during translocation by the ribosome. *eLife* **3**:e03406. doi: 10.7554/eLife.03406. Published 11 August 2014

In the published article, Kurt Fredrick's name was misspelt as ‘Kurt Fredrik’. Additionally, his contributions were given as ‘Drafting or revising the article, Contributed unpublished essential data or reagents’. They should have been given as ‘Conception and design, Contributed unpublished essential data or reagents’.

The article has now been corrected.

